# Discharge against medical advice among infants with 24–31 weeks’ gestation admitted to Chinese neonatal intensive care units: A multicenter cohort study

**DOI:** 10.3389/fped.2022.943244

**Published:** 2022-08-16

**Authors:** Wenlong Xiu, Ruimiao Bai, Xinyue Gu, Siyuan Jiang, Baoquan Zhang, Ya Ding, Yanchen Wang, Ling Liu, Jianhua Sun, Yun Cao, Wenhao Zhou, Shoo K. Lee, Zhankui Li, Changyi Yang

**Affiliations:** ^1^Department of Neonatology, Fujian Maternity and Child Health Hospital College of Clinical Medicine for Obstetrics and Gynecology and Pediatrics, Fujian Medical University, Fuzhou, China; ^2^Department of Neonatology, Northwest Women’s Children’s Hospital, Xi’an, China; ^3^NHC Key Laboratory of Neonatal Diseases (Fudan University), Children’s Hospital of Fudan University, Shanghai, China; ^4^Department of Neonatology, Children’s Hospital of Fudan University, Shanghai, China; ^5^Department of Neonatology, Guiyang Maternal Child Health Care Hospital, Guiyang, China; ^6^Department of Neonatology, Shanghai Children’s Medical Center, Shanghai Jiao Tong University School of Medicine, Shanghai, China; ^7^Maternal-Infant Care Research Centre and Department of Pediatrics, Mount Sinai Hospital, Toronto, ON, Canada; ^8^University of Toronto, Toronto, ON, Canada

**Keywords:** discharge against medical advice, preterm infants, mortality, morbidity, risk factor

## Abstract

**Background:**

Previous studies demonstrated high rates of discharge against medical advice (DAMA) among very preterm infants (VPIs) in China.

**Objectives:**

The aim of this study was to investigate the concurrent incidence, variation, and predictors of DAMA, along with the effect of DAMA on mortality of VPIs in China using data from the Chinese Neonatal Network (CHNN).

**Methods:**

All infants born at 24–31 completed weeks’ gestation and admitted to 57 CHNN neonatal intensive care units (NICUs) in 2019 were included for this cohort study, excluding infants with major congenital anomalies. Patient information was prospectively collected using the CHNN database. Multivariable log-linear regression analysis was used to assess the association of perinatal factors and DAMA.

**Results:**

A total of 9,442 infants born at 24–31 completed weeks’ gestation and admitted to 57 CHNN participating sites in 2019 were included in the study. Overall, 1,341 infants (14.2%) were discharged against medical advice. Rates of DAMA decreased with increasing gestational age (GA), and infants with lower GA were discharged earlier. DAMA infants had significantly higher rates of necrotizing enterocolitis, severe brain impairment, and bronchopulmonary dysplasia than non-DAMA infants. A total of 58.2% DAMA infants were predicted to die after discharge. The attributable risk percentage of mortality among DAMA infants was 92.4%. Younger maternal age, lower gestational age, small for gestational age, and Apgar score ≤3 at 5 min were independently associated with an increased risk of DAMA, while infants with antenatal steroids were less likely to be DAMA.

**Conclusion:**

The rate of DAMA in preterm infants between 24 and 31 weeks’ gestation remained high in China with a significant impact on the mortality rates. Continuous efforts to reduce DAMA would result in substantial improvement of outcomes for VPIs in China.

## Introduction

Infants born at less than 32 weeks (very preterm infants, VPIs) account for about 15–16% of all preterm births, with the highest mortality and morbidity rates (1, 2). In China and many other middle- or low-income countries, it has been reported that discharge against medical advice (DAMA) contributes significantly to the high mortality and morbidity of VPIs (3–8). DAMA refers to a situation wherein parents terminate treatment before the treating physicians recommend discharge. With limited physiological reserve of VPIs, they are at a very high risk of adverse outcomes in morbidity and mortality after DAMA (3, 4). Previous literature demonstrated that 70% of VPIs died following DAMA in China (9). Therefore, it is essential to collect detailed and high-quality data on DAMA of VPIs in China and to explore possible modified practices, aiming to reduce DAMA and improve overall outcomes of VPIs.

The Chinese Neonatal Network (CHNN) is a national collaborative research network focusing on neonatal and perinatal clinical studies in China (10). Established in 2018, CHNN has been maintaining a standardized database for all VPIs or very low birth weight infants admitted to NICUs across China from 1 January 2019. The objective of this study was to investigate the incidence, site variation, and predictors of DAMA, along with the impact of DAMA on mortality of VPIs in China using data from the CHNN.

## Materials and methods

### Study setting

This cohort study used the prospectively collected data from CHNN database. A total of 57 tertiary hospitals from 25 provinces and municipalities across China collected the whole year data using CHNN database in 2019, including all three national medical centers of children, all five regional medical centers of children, 30 provincial perinatal or children’s medical centers, and 19 major referral centers in large cities across China. Notably, 43 hospitals were perinatal centers with delivery facilities, and 14 hospitals were free-standing children’s hospitals. The ethics review board approved this study of Children’s Hospital of Fudan University (No. 2018-296), which all participating hospitals recognized. Waiver of consent was granted at all sites because of de-identified patient data.

The incidence of preterm birth in China was about 7.04% in 2015–2016 (11), with approximately 160,000 VPIs born in China each year (2). Therefore, CHNN database covered around 5% of VPIs who were born in China. VPIs not included in the database were either admitted to other NICUs or lower-level units, or not admitted to NICUs for treatment. Therefore, our data only represent outcomes of VPIs who received the highest level of care in major tertiary NICUs in China.

### Study design and population

All infants born at 24–31 completed weeks’ gestation and admitted to CHNN-participating neonatal intensive care units (NICUs) between 1 January 2019 and 31 December 2019 were eligible for this study. Infants with major congenital anomalies were excluded. Stillborn, delivery room deaths, moribund neonates on admission, and infants transferred to non-participating hospitals within 24 h after birth were not captured by the CHNN database. Re-admissions and transfers between participating hospitals were tracked as data from the same infants. Infants were followed until death or discharge.

### Data collection

Patient information was abstracted from patient charts by trained personnel using predefined standard definitions. Data were directly entered into a customized data entry program with built-in error checking. Data were subsequently sent electronically to the CHNN coordinating center located at the National Children’s Medical Center in Children’s Hospital of Fudan University in Shanghai, with patient identity kept confidential. Site investigator was responsible for data quality control in each site. The coordinating center checked data for quality and completeness and conducted audits to ensure the quality of data (12).

### Exposure

Discharge against medical advice, which is the exposure, was defined as when parents terminated treatment before the treating physicians recommended discharge according to discharge criteria in participating NICUs.

### Outcomes

Outcomes included mortality (primary outcome) and the five major neonatal morbidities, including necrotizing enterocolitis (NEC), severe brain injury, sepsis, bronchopulmonary dysplasia (BPD), and severe retinopathy of prematurity (ROP).

We did not follow up the DAMA infants after discharge and there were no other reliable sources of the information on the outcome of these infants. Therefore, we used predefined criteria to predict the likelihood of death for DAMA infants (13). If infants required invasive or non-invasive mechanical ventilation, inotropes infusion, or total parenteral nutrition (no enteral feeds initiated) on the day of discharge, we considered that they would not survive after discharge.

For morbidities, NEC was defined as stage ≥2 according to Bell’s stage (14, 15). Severe brain impairment was defined as degree ≥3 intraventricular hemorrhage (IVH) or cystic periventricular leukomalacia (cPVL). The diagnosis of severity of IVH was according to Papile’s criteria (16). cPVL was defined as the presence of periventricular cysts on cranial ultrasound or magnetic resonance imaging. Sepsis was defined as positive blood or cerebrospinal fluid culture (17). BPD was defined as ventilation or oxygen dependency at 36 weeks’ corrected age or at discharge, transfer, or death before 36 weeks (18). Severe ROP was defined as ROP stage III or above.

### Covariable definitions

Gestational age (GA) was determined using the hierarchy of best obstetric estimate based on prenatal ultrasound, menstrual history, obstetric examination, or all three. If the obstetric estimate was not available or was different from the postnatal pediatric estimate of gestation by more than 2 weeks, the GA was estimated using the Ballard score (19). Small for gestational age (SGA) was defined as birth weight <10th percentile for the GA according to the Chinese neonatal birth weight values (20). Prenatal care was defined as at least one pregnancy-related hospital visit during pregnancy. Antenatal steroids were defined as partial or complete courses of antenatal corticosteroids before birth.

### Statistical analysis

We described demographic characteristics by DAMA and non-DAMA group and provided crude risk ratios with 95% confidence interval to show the observed association between characteristics and DAMA. Kaplan–Meier curves were used to show the probability of non-DAMA over time after birth, while log-rank tests were applied to examine whether the probabilities were significantly different across GA groups. The Cochran–Armitage trend test was applied to examine the significance of the association between rate of DAMA and GA, and Jonckheere–Terpstra trend test was applied to determine the association between the median age at DAMA and GA. Attributable risk percent (ARP) was calculated to show the percentage of neonatal mortality that is predicted to be related to DAMA.

Multivariable log-linear regression models were used to measure the independent association between DAMA and perinatal factors. The model accounted for the cluster effect of different sites by applying generalized estimating equations.

### Missing data

The proportion of missing data were <10% for those variables included in the model and we believed that the data were not missing at random; therefore, we did not do multiple imputation. The data management and all statistical analyses were performed using SAS version 9.4 (SAS Institute, Inc., Cary, NC, United States). All analysis is done by SAS 9.4 software.

## Results

### Rates of discharge against medical advice

**FIGURE 1:**
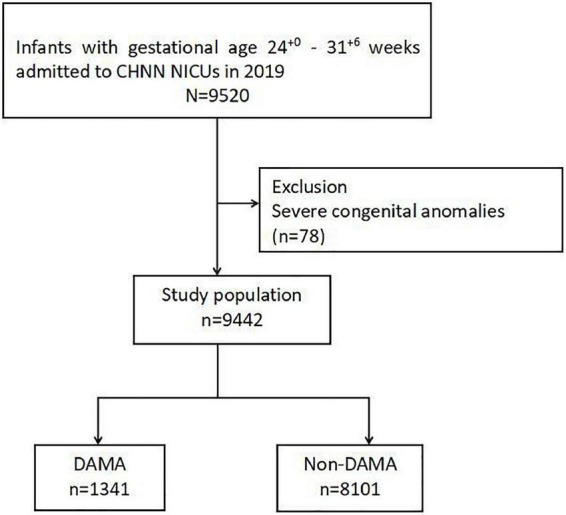
Study population. CHNN, Chinese Neonatal Network; DAMA, discharge against medical advice; NICU, neonatal intensive care unit.

During the study period, a total of 9,520 infants born at 24–31 completed weeks’ gestation were admitted to 57 CHNN sites, and 78 patients were excluded for major congenital anomalies. Thus, 9,442 cases were finally included in our study (Figure 1).

Overall, 1,341 infants (14.2%) were discharged against medical advice (Figure 1). Rates of DAMA decreased with increasing GA (Table 1). DAMA rates reached the peak of 43.5% at the GA of 24 weeks, while there were still 10.0% of infants born at 31 weeks discharged against medical advice.

**TABLE 1:** Rates of discharge against medical advice among infants born at 24^+0^–31^+6^ weeks’ gestation in Chinese NICUs by gestational age.

Gestational age (weeks)	Total number of infants	DAMA, *n* (%)*	Age at DAMA, *n* (%)^+^			
			
			Median (IQR)	≤3 days	4–14 days	>14 days
24	92	40 (43.5)	3 (1.5, 8.5)	21 (52.5)	12 (30.0)	7 (17.5)
25	228	67 (29.4)	6 (2, 20)	26 (38.8)	19 (28.4)	22 (32.8)
26	449	125 (27.8)	7 (1.5, 30.5)	50 (40.3)	29 (23.4)	45 (36.3)
27	837	178 (21.3)	11 (3, 51)	48 (27.0)	48 (27.0)	82 (46.0)
28	1,415	233 (16.5)	14 (3, 54)	68 (29.4)	48 (20.8)	115 (49.8)
29	1,763	202 (11.5)	22 (5, 46)	42 (20.9)	40 (19.9)	119 (59.2)
30	2,153	245 (11.4)	28.5 (7, 40.5)	37 (15.2)	45 (18.4)	162 (66.4)
31	2,505	251 (10.0)	20 (8, 35)	35 (14.0)	73 (29.2)	142 (56.8)
Total	9,442	1,341 (14.2)	16 (4, 41)	327 (24.5)	314 (23.5)	694 (52.0)

*Cochran–Armitage trend test for DAMA rate among GA group, as well as Jonckheere–Terpstra trend test for median age at DAMA among GA group, both have a significant *p*-value < 0.01.

^+^Six infants have missing value on their birth date, so that we cannot calculate their age at DAMA.

There was a huge variation of DAMA rates among different hospitals (Supplementary Figure 1). The range of DAMA rates among different hospitals was from 0 to 66.7% in 24–27 weeks infants, and 0.9–34.8% in 28–31 weeks infants.

### Age at discharge against medical advice

The median age at discharge for DAMA infants was 16 (IQR: 4–41) days. Age at discharge decreased with decreasing GA (Table 1 and Supplementary Figure 2). DAMA infants at 24 weeks were most likely to be discharged within 3 days after birth. In comparison, the majority of DAMA infants at 29–31 weeks were discharged after 14 days after birth.

### Maternal and infant characteristics

Baseline characteristics are summarized in Table 2. Infants in the DAMA group had lower GA and lower birth weight than infants who received complete care. They were also more likely to be female and outborn. Their mothers were younger, less likely to have maternal diabetes, and premature rupture of membranes. They were less likely to receive antenatal steroids and antenatal antibiotics and be delivered by cesarean section, compared with the non-DAMA group. Infants in the DAMA group were more likely to be SGA and had low Apgar scores (≤3) at 5 min.

**TABLE 2:** Baseline characteristics among infants born at 24^+0^–31^+6^ weeks’ gestation in China by DAMA and non-DAMA group.

	DAMA (*N* = 1,341)	Non-DAMA (*N* = 8,101)	Crude risk ratio (95% CI)
Maternal characteristics			
Maternal age, years, mean (SD)	29.9 (5.2)	31.0 (4.9)	0.96 (0.95, 0.97)*
Primigravida	655/1327 (49.4)	4,100/8,049 (51.0)	0.95 (0.86, 1.05)
Prenatal care	1,257/1,272 (98.8)	7,753/7,833 (99.0)	0.88 (0.55, 1.41)
Maternal hypertension	228/1,297 (17.6)	1,516/7,965 (19.0)	0.92 (0.80, 1.05)
Maternal diabetes	187/1,292 (14.5)	1,397/7,952 (17.6)	0.82 (0.71, 0.95)
Premature rupture of membranes	705/1,245 (56.6)	4,557/7,589 (60.1)	0.89 (0.80, 0.98)
Antenatal antibiotics	440/1,066 (41.3)	3,137/6,791 (46.2)	0.84 (0.75, 0.94)
Antenatal steroids	736/1,156 (63.7)	5,711/7,355 (77.7)	0.56 (0.50, 0.63)
Cesarean delivery	597/1,335 (44.7)	4,558/8,059 (56.6)	0.67 (0.60, 0.74)
Infant characteristics			
Gestational age, weeks, median (IQR)	29.0 (27.6, 30.6)	30.0 (28.6, 31.0)	0.81 (0.79, 0.84)*
Birth weight, grams, mean (SD)	1,208.5 (340.0)	1,343.0 (311.0)	0.89 (0.87, 0.90)*^+^
Small for gestational age	123/1,341 (9.2)	5,18/8,101 (6.4)	1.39 (1.17, 1.64)
Outborn	539/1,341 (40.2)	2,883/8,101 (35.6)	1.18 (1.07, 1.31)
Female	614/1,341 (46.0)	3,472/8,101 (42.9)	1.11 (1.01, 1.23)
Multiple birth	397/1,341 (29.6)	2,427/8,101 (30.0)	0.99 (0.88, 1.10)
Apgar score ≤3 at 5 min	38/1,216 (3.1)	77/7,626 (1.0)	2.45 (1.88, 3.19)

DAMA, discharge against medical advice; SD, standard deviation; IQR, interquartile range.

The denominator for certain rate is the number of infants with certain medical record, which means some data have missing values; DAMA was set as an event, while non-DAMA was set as a control.

*Mean risk ratio for continuous variables, with 1-unit increase if not specified (ratio between probability of observing event = 1 when exposure = x + 1 over exposure = x).

^+^With 100-unit (gram) increase.

### Morbidities and mortality among discharge against medical advice infants

Discharge against medical advice infants had higher rates of neonatal morbidities, including NEC, severe brain impairment, and BPD, compared with infants who received complete care (Table 3 and Supplementary Table 1). Overall, 58.2% (781/1,341) of DAMA infants required at least one type of intensive treatment at the time of discharge; hence, they were predicted to die after discharge (Supplementary Table 2). The predicted mortality among DAMA infants was 80.5 and 48.4% for infants <28 and 28–31 weeks, which was significantly higher than the observed mortality among infants who received complete care (14.2 and 6.4%, respectively) (Table 4).

**TABLE 3:** Major neonatal morbidities among infants born at 24^+0^–31^+6^ weeks’ gestation in China by DAMA and non-DAMA group.

Morbidities	DAMA (*N* = 1,341)	Non-DAMA (*N* = 8,101)	Crude risk ratio (95% CI)
NEC stage II or above	91/1,341 (6.8)	401/8,101 (5.0)	1.32 (1.09, 1.61)
Severe brain impairment*	189/818 (23.1)	735/7,138 (10.3)	2.29 (1.97, 2.65)
IVH grade III or above	148/808 (18.3)	473/7,098 (6.1)	2.63 (2.25, 3.08)
cPVL	83/881 (9.4)	386/7,416 (5.2)	1.74 (1.41, 2.13)
Sepsis	107/1,341 (8.0)	752/8,101 (9.3)	0.87 (0.72, 1.04)
Early-onset sepsis	21/1,341 (1.6)	109/8,101 (1.4)	1.14 (0.77, 1.69)
Late-onset sepsis	88/1,071 (8.2)	658/7,948 (8.3)	0.82 (0.67, 1.00)
ROP stage III or above^+^	21/490 (4.3)	293/6,803 (4.3)	1.00 (0.65, 1.52)
BPD at corrected GA 36 week ^#^	168/321 (52.3)	1,737/4,802 (36.2)	1.85 (1.50, 2.29)

NEC, necrotizing enterocolitis; IVH, intraventricular hemorrhage; cPVL, cystic periventricular leukomalacia; ROP, retinopathy of prematurity; BPD, bronchopulmonary dysplasia. The denominator for certain rate is the number of infants with certain medical record, which means some data have missing values; DAMA was set as an event, while non-DAMA was set as a control.

*Severe brain impairment is defined as severe IVH (grade 3 or 4) and/or cPVL, whose rate is calculated among infants with neuroimaging results.

^+^Rate of severe ROP is calculated among infants with ROP screening result.

^#^Rate of BPD is calculated among infants staying in NICU till their corrected GA reached 36 weeks.

**TABLE 4:** Neonatal mortality of DAMA and non-DAMA infants born at 24^+0^–31^+6^ weeks’ gestation in China.

Gestational age (weeks)	Overall death, *n*/*N* (%)	In-hospital death among non-DAMA, *n*/*N* (%)*	Predicted death among DAMA, *n*/*N* (%)^+^	ARP (95% CI), %^#^
24	61/92 (66.3)	23/52 (44.2)	38/40 (95.0)	53.5 (52.8, 54.1)
25	103/228 (45.2)	40/161 (24.8)	63/67 (94.0)	73.6 (73.1, 74.1)
26	137/449 (30.5)	39/324 (12.0)	98/125 (78.4)	84.7 (84.3, 85.0)
27	199/837 (23.8)	68/659 (10.3)	131/178 (73.6)	86.0 (85.7, 86.3)
28	216/1,415 (15.3)	72/1,182 (6.1)	144/233 (61.8)	90.1 (89.9, 90.4)
29	158/1,763 (9.0)	47/1,561 (3.0)	111/202 (55.0)	94.5 (94.3, 94.8)
30	140/2,153 (6.5)	34/1,908 (1.8)	106/245 (43.3)	95.9 (95.6, 96.1)
31	123/2,505 (4.9)	33/2,254 (1.5)	90/251 (35.9)	95.9 (95.7, 96.1)
Total	1,137/9,442 (12.0)	356/8,101 (4.4)	781/1,341 (58.2)	92.5 (92.4, 92.6)

*In-hospital death among non-DAMA are exactly based on medical record. Infants reported “Died” or “Palliative Care” were counted as death cases, while other situations were regarded as survival. Death among DAMA was defined as receiving intensive care at discharge, as shown in Table 4.

^+^Defined as requiring invasive or non-invasive mechanical ventilation, inotropes infusion, or total parenteral nutrition (no enteral feeds initiated) on the day of discharge.

^#^APR, attributable risk percent = [(p1 − p2)/p1] × 100 = [(Risk Ratio − 1)/Risk Ratio] × 100 (alternatively).

Overall, the attributable risk percentage of mortality was 92.4% among DAMA VPIs. The attributable risk percentage of DAMA on mortality increased with advancing GA (Table 4).

### Risk factors for discharge against medical advice

Younger mother, lower GA (24–28 weeks), SGA, and Apgar score ≤3 at 5 min were independently associated with an increased risk of DAMA. Infants who received antenatal steroids were less likely to be DAMA (Table 5).

**TABLE 5:** Perinatal factors associated with DAMA among infants born at 24^+0^–31^+6^ weeks’ gestation in China.

Characteristics	Adjusted risk ratio (95% CI)^#^
Maternal characteristics*	
Maternal age, years^+^	0.97 (0.96, 0.98)
Maternal diabetes	0.90 (0.75, 1.08)
Premature rupture of membranes	0.90 (0.76, 1.07)
Antenatal steroids	0.70 (0.60, 0.83)
Cesarean delivery	0.93 (0.81, 1.06)
Infant characteristics*	
Gestational age, weeks	
24	4.83 (3.37, 6.92)
25	3.28 (2.32, 4.63)
26	3.13 (2.36, 4.16)
27	2.43 (1.90, 3.12)
28	1.79 (1.51, 2.14)
29	1.10 (0.93, 1.31)
30	1.18 (0.96, 1.44)
31	reference
Small for gestational age	1.76 (1.49, 2.07)
Outborn	1.11 (0.87, 1.41)
Female	1.09 (0.95, 1.24)
Apgar score ≤3 at 5 min	1.61 (1.12, 2.31)

NEC, necrotizing enterocolitis; ROP, retinopathy of prematurity; BPD, bronchopulmonary dysplasia.

Non-DAMA set as control.

*Those maternal and infant characteristics with p-value less than 0.05 in Table 2 are selected into the multivariate model, except for antenatal antibiotics and birth weight because of their collinearity with antenatal steroids and gestational age respectively.

^+^For maternal age as a continuous variable, odds ratios correspond to 1-year increase.

^#^The risk ratios (RR) and 95% CI were obtained by an extension of multivariable log-linear regression model, accounting for cluster effect of different sites by means of generalized estimating equations (GEE). The RR for each variable was adjusted for other variables listed in the table.

## Discussion

To the best of our knowledge, our report is the largest and latest nationwide study focusing on DAMA of VPIs in China. We identified a high rate of DAMA and substantial variation among different hospitals among VPIs in China. DAMA infants had high rates of severe brain impairment, BPD, and NEC, and showed a significant impact on mortality of VPIs. Younger maternal age, lower GA, SGA, and Apgar score ≤3 at 5 min were independently associated with an increased risk of DAMA, while antenatal steroids were associated with a decreased risk of DAMA.

Previous studies indicated near 21% of neonates with respiratory failure were DAMA in China in 2007–2008 (6–8). Jiang reported that the incidence of DAMA among VPIs in China was 19.7% in 2015–2016 and 13.3% in 2015–2018 (3, 13). Given the observed 14.2% DAMA rates among VPIs in our study, it has to be acknowledged that the rate of DAMA in preterm infants between 24 and 31 weeks’ gestation remained high in China. Therefore, targeted efforts are definitely needed to reduce further or eliminate DAMA.

One improvement target may focus on infants with relatively larger GA. There were still 10–11% of infants born at 31 and 30 weeks discharged prematurely, resulting in high mortality for these infants. Given the large proportion of these infants among all VPIs admitted to Chinese NICUs, reducing DAMA rates among infants at 30–31 weeks will result in a significant reduction of the absolute number of DAMA.

The in-hospital mortality rate of VPIs with complete care in our research was close to that in developed countries, such as the Canada Neonatal Network (CNN) in the same period (4.4% in CHNN vs. 4.8% in CNN) (21). However, our data confirmed that the in-hospital mortality rates for VPIs in China should be interpreted with caution due to the high rate of DAMA and high mortality among DAMA infants. After taking DAMA infants into consideration, the overall mortality rates almost tripled. Also, DAMA infants showed higher rates of major neonatal morbidities. Therefore, when monitoring outcomes longitudinally or comparing outcomes regionally, nationally, or internationally, the DAMA infants should be taken into consideration to avoid overly optimistic estimates.

The attributable risk percentage of mortality was 92.4% among DAMA VPIs in our study, which means that 92.4% of deaths among DAMA infants might be related to the DAMA decision. However, these data should be interpreted with caution. In many instances, DAMA is likely to be motivated by a poor prognosis with regard to impending death or serious disability. Therefore, the overall attributable risk percentage of 92.4% might overestimate the proportion of deaths due to the DAMA decision. Anyhow, efforts to reduce DAMA rates will substantially decrease the mortality of VPIs in Chinese NICUs. Also, one cohort study estimated potential outcomes of DAMA among VPIs in China if they receive complete care based on outcomes of a group of propensity score matched non-DAMA infants. The studies found that 59% DAMA infants aged <32 weeks might have intact survival if intensive care services are provided (13).

Our data showed that infants with the youngest GA were mostly discharged within 3 days after birth, which indicated that parents tended to make an early decision of DAMA for infants with lower GA. Their discharge might be due to parents’ concerns about the continuing suffering and grave outcomes, in terms of death or adverse neurodevelopmental outcomes. It probably reflected their lack of confidence in the chance of survival and outcome of infants born at lower GA both from parents and health providers. However, in recent decades, there have been major advances in the survival rate and outcome of infants with the smallest GAs in China (3, 10, 13). Obstetricians and neonatologists need to be aware of the latest data and inform families to make appropriate choices. For those infants with higher GA (29–31 weeks), their discharge might be associated with neonatal morbidities, which might be a serious blow for parents, giving most of them discharged after 14 days of life. Our data demonstrated that more infants in the DAMA group had severe brain impairment than the non-DAMA group, suggesting that fears for poor long-term outcomes might be one of the major reasons parents terminate the treatment of their babies. There were also more NEC and BPD in the DAMA group than in the non-DAMA group in VPIs. Therefore, more effort should be made to reduce those morbidities in VPIs. Our study also identified that perinatal factors, including low GA, SGA, younger mother, and low Apgar score at 5 min, were associated with an increased risk of DAMA, while antenatal steroids served as a protective factor for DAMA. Appropriate perinatal care may also contribute to the reduction of DAMA.

According to the published literature, besides concern about poor prognosis, the inability to afford the cost might also be a major reason for DAMA (22–24). However, increasing coverage of health insurance for neonates across China, change of one-child policy, and improving neonatal intensive care may result in changes in decision making of DAMA. Unfortunately, we did not collect information on the reasons for DAMA in our study. However, by recognizing this remaining problem, CHNN has launched targeted quality improvement initiatives aiming to reduce DAMA, with the first step as a prospective survey on specific DAMA reasons.

The development of neonatology and neonatal intensive care has been uneven among different districts in China. As found in our study, previous reports also showed significant variation of mortality and DAMA among VPIs in different hospitals in China (3, 4). Besides the variations in medical practice, there are various reasons for this wide variation of DAMA rates among different NICUs in China, which might include the sociocultural, socioeconomic, religious, and ethnic differences. Unfortunately, we did not collect this relevant information, so we were unable to perform a rigorous analysis of the causes of this variation. Recognizing these remaining problems, relevant studies organized by CHNN are underway. And a national network-level collaborative, a multifaceted quality improvement approach may be helpful to decrease the rate of DAMA *via* benchmarking, best-practice consensus, and sharing expertise.

Our study has several strengths. Our data were from the multicenter standardized database with clear definitions of variables and strict quality control, which reduced information bias. All participating NICUs were large tertiary centers representing the highest level of neonatal care in representing areas of China, which made the comparison of data reasonable. The large sample size enabled detailed stratification of GA, which can provide health providers and policymakers a more precise reference at each week of GA.

There were several limitations in our study. First, this is a hospital-based study instead of a population-based study. Delivery room deaths and live births without active resuscitation who were never admitted to NICUs were not included. This would result in an underestimation of the DAMA rate. Second, we did not actually follow up the DAMA infants after discharge, so we could not obtain an accurate mortality rate for these infants. Third, we could not identify specific reasons for DAMA and the causes of variability of DAMA rates among different NICUs because the database did not include such information.

## Conclusion

Discharge against medical advice among VPIs has been decreasing over the past few years but remained high. DAMA had a significant impact on the mortality of VPIs in China. Continuous efforts to reduce DAMA would result in substantial improvement of outcomes for VPIs in China.

## Members of the Chinese Neonatal Network

Chairmen: Chao Chen, Children’s Hospital of Fudan University; Shoo K. Lee, Mount Sinai Hospital, University of Toronto. Vice-Chairmen: Lizhong Du, Children’s Hospital of Zhejiang University School of Medicine; Wenhao Zhou, Children’s Hospital of Fudan University. Site principle investigators of the Chinese Neonatal Network: Children’s Hospital of Fudan University: Yun Cao; The Third Affiliated Hospital of Zhengzhou University: Falin Xu; Tianjin Obstetrics & Gynecology Hospital: Xiuying Tian; Guangzhou Women and Children’s Medical Center: Huayan Zhang; Children’s Hospital of Shanxi: Yong Ji; Northwest Women’s and Children’s Hospital: Zhankui Li; Gansu Provincial Maternity and Child Care Hospital: Jingyun Shi; Shengjing Hospital of China Medical University: Xindong Xue; Shenzhen Maternity and Child Health Care Hospital: Chuanzhong Yang; Quanzhou Women and Children’s Hospital: Dongmei Chen; The Affiliated Suzhou Hospital of Nanjing Medical University: Sannan Wang; Guizhou Women and Children’s Hospital/Guiyang Children’s Hospital: Ling Liu; Hunan Children’s Hospital: Xirong Gao; The First Bethune Hospital of Jilin University: Hui Wu; Fujian Maternity and Child Health Hospital, Affiliated Hospital of Fujian Medical University: Changyi Yang; Nanjing Maternity and Child Health Care Hospital: Shuping Han; Qingdao Women and Children’s Hospital: Ruobing Shan; The Affiliated Hospital of Qingdao University: Hong Jiang; Children’s Hospital of Shanghai: Gang Qiu; Women and Children’s Hospital of Guangxi Zhuang Autonomous Region: Qiufen Wei; Children’s Hospital of Nanjing Medical University: Rui Cheng; Henan Children’s Hospital: Wenqing Kang; The First Affiliated Hospital of Xinjiang Medical University: Mingxia Li; Foshan Women and Children’s Hospital: Yiheng Dai; The First Affiliated Hospital of Anhui Medical University: Lili Wang; Shanghai First Maternity and Infant Hospital: Jiangqin Liu; Yuying Children’s Hospital Affiliated to Wenzhou Medical University: Zhenlang Lin; Children’s Hospital of Chongqing Medical University: Yuan Shi; The First Affiliated Hospital of Zhengzhou University: Xiuyong Cheng; The First Affiliated Hospital of USTC, Division of Life Sciences and Medicine, University of Science and Technology of China: Jiahua Pan; Shaanxi Provincial People’s Hospital: Qin Zhang; Children’s Hospital of Soochow University: Xing Feng; Wuxi Maternity and Child Healthcare Hospital: Qin Zhou; People’s Hospital of Xinjiang Uygur Autonomous Region: Long Li; The Second Xiangya Hospital of Central South University: Pingyang Chen; Qilu Children’s Hospital of Shandong University: Xiaoying Li; Hainan Women and Children’s Hospital: Ling Yang; Xiamen Children’s Hospital: Deyi Zhuang; Xinhua Hospital Affiliated to Shanghai Jiao Tong University School of Medicine: Yongjun Zhang; Shanghai Children’s Medical Center, Shanghai Jiao Tong University School of Medicine: Jianhua Sun; Shenzhen Children’s Hospital: Jinxing Feng; Children’s Hospital Affiliated to Capital Institute of Pediatrics: Li Li; Women and Children’s Hospital, School of Medicine, Xiamen university: Xinzhu Lin; General Hospital of Ningxia Medical University: Yinping Qiu; First Affiliated Hospital of Kunming Medical University: Kun Liang; Hebei Provincial Children’s Hospital: Li Ma; Jiangxi Provincial Children’s Hospital: Liping Chen; Fuzhou Children’s Hospital of Fujian Province: Liyan Zhang; First Affiliated Hospital of Xian Jiao Tong University: Hongxia Song; Dehong People’s Hospital of Yunnan Province: Zhaoqing Yin; Beijing Children’s Hospital, Capital Medical University: Mingyan Hei; Zhuhai Center for Maternal and Child Health Care: Huiwen Huang; Guangdong Women and Children’s Hospital: Jie Yang; Dalian Municipal Women and Children’s Medical Center: Dong Li; Peking Union Medical College Hospital: Guofang Ding; Obstetrics & Gynecology Hospital of Fudan University: Jimei Wang; Shenzhen Hospital of Hongkong University: Qianshen Zhang; Children’s Hospital of Zhejiang University School of Medicine: Xiaolu Ma; Advisor: Joseph Ting; University of Alberta.

## Data availability statement

The datasets for this article are not publicly available because part of the data is included in another article under preparation. Requests to access the datasets should be directed to YC, yuncao@fudan.edu.cn.

## Ethics statement

The studies involving human participants were reviewed and approved by the Ethics Committee of the Children’s Hospital of Fudan University. Written informed consent from the participants’ legal guardian/next of kin was not required to participate in this study in accordance with the national legislation and the institutional requirements.

## Author contributions

WX, RB, ZL, and CY: study concept and design. WX, RB, XG, SJ, YW, LL, JS, YC, WZ, SL, ZL, and CY: acquisition, analysis, and interpretation of data. WX, RB, XG, and SJ: drafting of manuscripts. All authors: critical revision of the manuscripts for important intellectual content. XG and SJ: statistical analysis. SL: obtained the funding. BZ, YD, LL, JS, YC, WZ, ZL, and CY: administrative and technical or maternal support. JS, YC, WZ, and SL: supervision.
