# Supplementary material for: Discharge against medical advice among infants with 24–31 weeks’ gestation admitted to Chinese neonatal intensive care units: A multicenter cohort study
Source: Front Pediatr. 2022 Aug 16;10:943244. doi: 10.3389/fped.2022.943244 (PMC9424540; doi:10.3389/fped.2022.943244)
Supplement: Supplementary file 1 [file Data_Sheet_1.docx]

**Supplementary Figure 1, Rates and 95%C.I. of DAMA by Sites, stratified by gestational age weeks (24^+0^ - 27^+6^ ; 28^+0^ - 31^+6^)**

**
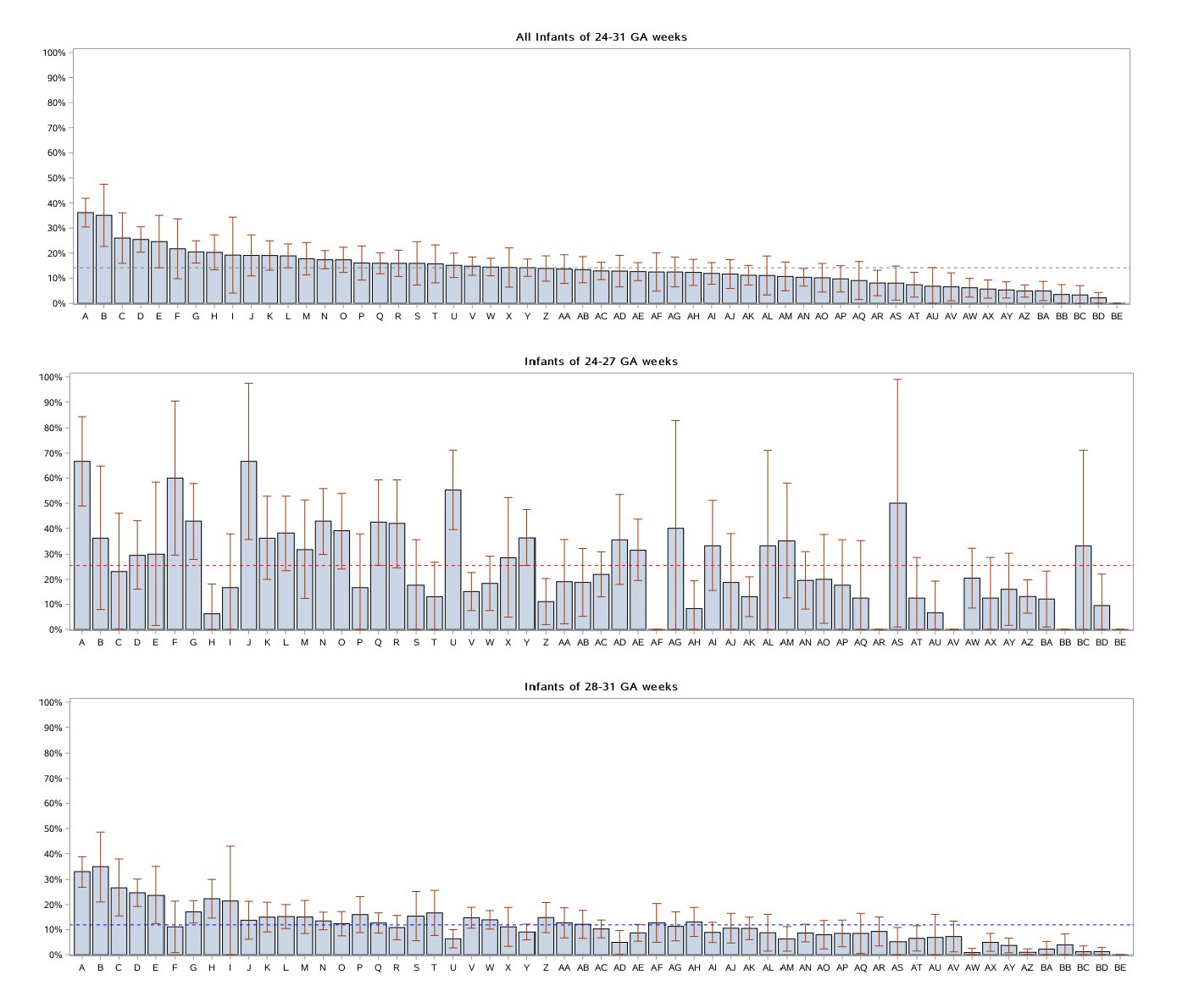
**

**Note: Site ID is anonymous and the bars are in order of DAMA rate among all infants of 24^+0^ - 31^+6^ GA weeks. Grey line is the average rate (14.2%) of the study population, while the red dashed line (25.5%) is for 24^+0^ - 27^+6^ weeks’ infants and blue dashed line (11.9%) is for 28^+0^ - 31^+6^ weeks’ infant. The letters for each hospital the same across the three panels.**

**Supplementary Figure 2,** **Kaplan-Meier curves regarding the time of DAMA for infants with different gestational weeks**

**
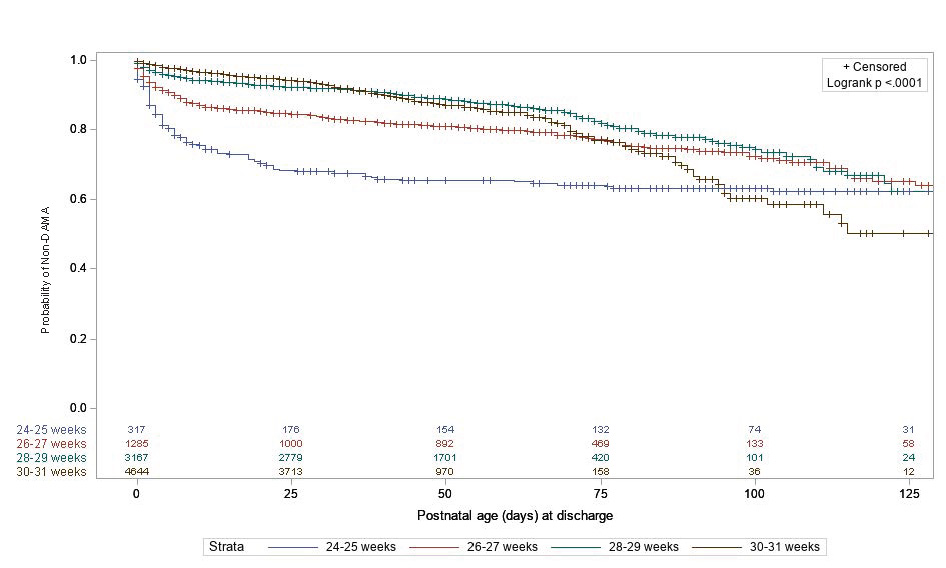
**

**Supplementary Table 1, Major neonatal morbidities among infants born at 24^+0^ - 31^+6^ weeks’ gestation in China by DAMA and non-DAMA group, stratified by gestational age week**

| **Morbidities, , n/N (%)** | **24-25 weeks (N=320)** | | **26-27 weeks (N=1280)** | | **28-29 weeks (N=3178)** | | **30-31 weeks (N=4658)** | |
| --- | --- | --- | --- | --- | --- | --- | --- | --- |
|  | **DAMA** | **non-DAMA** | **DAMA** | **non-DAMA** | **DAMA** | **non-DAMA** | **DAMA** | **non-DAMA** |
| NEC | 7/107 (6.5) | 21/213 (9.7) | 15/303 (5.0) | 64/983 (6.5) | 31/435 (7.1) | 137/2743 (5.0) | 38/496 (7.7) | 179/4162 (4.3) |
| Sepsis | 11/107 (10.3) | 38/213 (17.8) | 34/303 (11.2) | 127/983 (12.9) | 37/435 (8.5) | 318/2743 (11.6) | 25/496 (5.0) | 269/4162 (6.5) |
| Severe brain impairment | 19/56 (33.9) | 44/168 (26.2) | 61/179 (34.1) | 144/853 (16.9) | 59/264 (22.4) | 270/2455 (11.0) | 50/319 (15.7) | 277/3662 (7.6) |
| Severe ROP | 0/11 (0.0) | 42/146 (28.8) | 9/88 (10.7) | 90/852 (10.6) | 7/163 (4.3) | 99/2438 (4.1) | 5/228 (2.2) | 62/3367 (1.8) |
| BPD at corrected GA 36 week | 4/6 (66.7) | 88/126 (69.8) | 41/55 (74.6) | 363/687 (52.8) | 55/110 (50.0) | 703/1687 (41.7) | 68/150 (45.3) | 583/2302 (25.3) |

**Supplementary Table 2, Intensive care at discharge among DAMA infants** **born at 24^+0^ - 31^+6^ weeks’ gestation in China**

| **Gestational age (weeks)** | **Total number of DAMA infants** | **Any intensive care, n (%)** | **Invasive ventilation, n (%)** | **Non-invasive ventilation, n (%)** | **Inotropes, n (%)** | **TPN,**  **n (%)** |
| --- | --- | --- | --- | --- | --- | --- |
| 24 | 40 | 38 (95.0) | 31 (77.5) | 5 (12.5) | 14 (35.0) | 20 (50.0) |
| 25 | 67 | 63 (94.0) | 45 (67.2) | 16 (23.9) | 17 (25.4) | 28 (41.8) |
| 26 | 125 | 98 (78.4) | 68 (54.4) | 29 (23.2) | 26 (20.8) | 46 (36.8) |
| 27 | 178 | 131 (73.6) | 96 (53.9) | 36 (20.2) | 45 (25.3) | 54 (30.3) |
| 28 | 233 | 144 (61.8) | 94 (40.3) | 47 (20.2) | 42 (18.0) | 74 (31.8) |
| 29 | 202 | 111 (55.0) | 67 (33.2) | 43 (21.3) | 27 (13.4) | 48 (23.8) |
| 30 | 245 | 106 (43.3) | 61 (24.9) | 38 (15.5) | 31 (12.7) | 49 (20.0) |
| 31 | 251 | 90 (35.9) | 44 (17.5) | 33 (13.2) | 23 (9.2) | 45 (17.9) |
| Total | 1341 | 781 (58.2) | 506 (37.7) | 247 (18.4) | 225 (16.8) | 364 (27.1) |
